# Does anyone need help? Age and gender effects on children's ability to recognize need-of-help

**DOI:** 10.3389/fpsyg.2014.00170

**Published:** 2014-02-27

**Authors:** Margarita Stolarova, Aenne A. Brielmann

**Affiliations:** ^1^Department of Psychology and Zukunftskolleg, University of KonstanzKonstanz, Germany; ^2^Department of Society and Economics, Rhine-Waal University of Applied SciencesKleve, Germany

**Keywords:** child development, social categorization, helping behavior, prosocial behavior, altruism, socioemotional development, psychology, gender differences

## Abstract

The exploratory study presented here examines children's ability to recognize another person's need-of-help. This social perception process necessarily precedes the decision to actively help others. Fifty-eight children aged between 5 and 13 completed three experimental paradigms. They were asked to look at black-and-white drawings and to indicate which ones showed somebody in need of help. A control task requiring children to differentiate between pictures of humans and birds measured general categorization abilities. This experimental design enabled us to consider confounding effects of children's developmental status and motivation and to distinguish them from specific need-of-help recognition abilities. As gender and age have been shown to influence social perception as well as helping behavior, we explored whether these factors also have an impact on need-of-help recognition. Children's response accuracies and response times (RTs) were analyzed. We observed clearly higher accuracy rates for younger girls compared to younger boys specifically in the need-of-help recognition tasks. For boys, an age-related performance improvement was found. Younger girls performed at a similarly high level as older girls and boys. No gender differences were observed for children aged over nine. This report provides first evidence that the developmental trajectory of children's ability to recognize another person's need-of-help differs for girls and boys.

## Introduction

Helping is an important aspect of prosocial interaction in humans and to a lesser degree in primates (Warneken and Tomasello, 2006, 2007; Liebal et al., 2008; Dunfield and Kuhlmeier, 2010; Koski and Sterck, 2010). In recent years, developmental psychology has seen a rise in the assessment of prosocial behavior in infants and toddlers. Many studies have documented children's early willingness and ability to help, (Hamlin et al., 2007; Dunfield and Kuhlmeier, 2010; Svetlova et al., 2010; Vaish et al., 2010; Hepach et al., 2012, 2013b; Carpenter et al., 2013), even preverbal toddlers exhibit clear tendencies to actively help others (Liszkowski et al., 2006; Warneken and Tomasello, 2007, 2009). Moreover, infants as young as 6 months seem to prefer the “helper” over the “hinderer” (Hamlin et al., 2007) and the “victim” over the “aggressor” (Kanakogi et al., 2013). In a recent paper reviewing three separate experiments Hepach et al. (2013a) argued that toddlers and infants are intrinsically motivated to help others. The authors see the underlying mechanism for this motivational pattern and the following active helping in evolutionary selection for prosocial behavior within a group of interdependent individuals. A few other studies have considered need-of-help recognition in the framework of Theory of Mind or attribution of intention (e.g., Brunet et al., 2000; Völlm et al., 2006. All these studies assume that infants and young children understand when someone needs help in given (experimental) situations. However, both the motivation to help and active helping are necessarily preceded by perceptual processes. The realization that a person wants to but cannot achieve a certain goal has to occur before someone can be motivated to help this person. Thus, while the studies briefly reviewed above are very insightful when it comes to children's helping motivation, active helping abilities and their development, they do not assess an important perceptual precondition for active helping: need-of-help recognition. While it can be assumed that need-of-help has been recognized when helping does occur, the reverse inference cannot be made. This study explores children's ability to recognize need-of-help. In this way, it contributes to the discussion on different stages of prosocial action by separating children's need-of-help recognition abilities from later stages, such as motivational processes, decisions, and active helping that have been considered before (e.g., Dunfield and Kuhlmeier, 2010; Hepach et al., 2012, 2013b). This study thus provides a starting point for investigating the limits and determinants of need-of-help recognition as a specific process of social perception. We derived the variables of interest to our analyses from research assessing active helping behavior and its underlying motivation, as well as from studies investigating social perception, since the present report is explorative and the first to investigate children's need-of-help recognition abilities in a controlled experimental setting.

One factor which has been found to influence human's tendency to help and prosocial behavior in general is gender^1^ (for a review see Eagly, 2013). Girls are rated higher on different measures of prosocial behavior and empathy (e.g., using parent and teacher questionnaires) including the tendency to help (e.g., Malti et al., 2009; Ensor et al., 2011). A higher amount of helping and cooperation for girls compared to boys has also been found in some (e.g., Kirschner and Tomasello, 2010: Ensor et al., 2011), but not in all empirical reports on children's active helping behavior in experimental settings (e.g., Renouf et al., 2010; Brownell et al., 2013). Similarly, in the literature on adults, we find diverging results (Croson and Gneezy, 2009; Eagly, 2009; Balliet et al., 2011; for a recent overview on gender differences in social behavior see Eagly, 2013). As gender differences may stem from a broad range of biological and social causes, as well as their complex interactions, it is not surprising that findings regarding gender influences on different types of active helping are ambiguous. Investigating the socio-perceptual processes preceding helping behavior in children could help to disambiguate conflicting findings in this domain. Systematic gender differences in social perception have been observed in studies with adult participants (Proverbio et al., 2008, 2010), and also in studies that include children in their scope (Anderson et al., 2013). These reports found that female participants show greater brain activation for social stimuli vs. non-social stimuli compared to male participants of different ages. Building upon these findings on gender differences in active helping, social perception and their underlying neuropsychological mechanisms, we have explored whether the ability of boys and girls to recognize another person's of need of help differs, too. On one hand, previous literature regarding social perception puts forward greater amount of helping and higher resource allocation toward socially relevant information in women. Based on these findings, one could expect girls to show superior need-of-help recognition. On the other hand, previous results regarding differences between male and female participants in active helping are mixed, suggesting that need of help recognition might be equally good for both genders.

Differences between adult men and women regarding helping behavior have also been shown to depend to a certain extent on the gender of the person receiving help (Eagly, 2009): Men tend to help women more often than women do, albeit that this finding is restricted to situations in which others are watching. Moreover, neural correlates of socio-perceptual processes have also been reported to be partially own-gender specific with later stages of processing showing greater activation for neutral own-gender faces compared to neutral other-gender faces (for a recent review see Kret and De Gelder, 2012). We included pictures of boys and girls in our stimuli to investigate the potential influence of the depicted person's gender on children's need-of-help recognition.

Previous research has also demonstrated that even though children are motivated and able to help others early in life (e.g., Liszkowski et al., 2006; Hamlin et al., 2007; Warneken and Tomasello, 2007; Svetlova et al., 2010) the amount of active helping increases with age (e.g., Cassidy et al., 2003). Several variables have been shown to contribute to this increase in prosocial behavior, e.g., verbal abilities, executive functions in general as well as emotion regulation (e.g., Cassidy et al., 2003; Cole et al., 2010; Ensor et al., 2011; Miyake and Friedman, 2012; Monopoli and Kingston, 2012; for an overview see Decety, 2010). The fact that the quantity of children's helping behavior increases with age might also be attributed to specific aspects of socioemotional development. Anderson et al. (2013) report that the greater activity and connectivity in response to viewing biological motion as opposed to random motion also increases with age amid female participants. Our study aimed to clarify whether the capacity to recognize another person's need-of-help changes with age. We chose a population of children old enough to understand and perform the tasks posed so as to evaluate children's explicit responses, i.e., accuracy rates and response times (RTs). We also assessed whether there is a systematic interaction between a child's gender and their age with regard to need-of-help recognition. Therefore, we divided our convenience sample into three age groups and included both age and gender as possible influential factors.

In order to assess whether expected effects of age and gender are specific to need-of-help recognition and are not generalizable to categorization tasks *per se*, we designed three mutually controlling experimental paradigms. In all of them the identical set of visual stimuli was used, but they differed in terms of instruction, task and experimental design (see section “Experimental design and procedure” and Figure 2 for details). Need-of-help recognition was operationalized through children's response accuracy (hit rates). Children's RTs served to assess ease of processing and decision making. In one of the three paradigms, children were asked to indicate whether the picture they had just briefly seen showed someone in need of help or not (“NoH-distinction”). In the second help-content related paradigm, the question posed was which one out of two pictures presented side-by-side showed someone in need of help (“NoH-comparison”). The control paradigm (“Human-bird-distinction”) used the same design as the first need-of-help recognition paradigm. Here, however, children were asked to indicate whether they had just seen a bird or a human and need-of-help content was irrelevant. This last paradigm allowed us to monitor unspecific effects of motivation and development. Additional insights regarding the possible influences of motivation on task performance were provided by comparing the children who were able and willing to complete all three paradigms with those children who only finished one or two tasks.

The present study contributes to the discussion of gender and age related differences with regard to helping and social perception by investigating need-of-help recognition. We separated this social perception process preceding motivational and decisional aspects of helping behavior, in order to shed light on the preconditions for active helping. We aimed to answer the following questions: Does need-of-help recognition improve with age or is it accomplished at an early stage of development? Are there differences between boys and girls regarding need-of-help recognition? If there are gender related differences, how do these change with age and do they depend on the gender of the depicted child?

## Methods

### Ethics statement

Parents gave written informed consent according to the principles of the Declaration of Helsinki before their children participated in this study. Special care was taken to ensure that parents and children understood that their participation was voluntary and could be ended at any time.

### Participants and exclusion criteria

Data collection for this study occurred at an open-air cost-free festival for children that took place in the German city of Konstanz. For every participant, minimal demographic information was obtained: age, gender, and ownership of pets or not. No child was refused participation and no pre-selection of any kind took place. This resulted in a relatively broad convenience sample with regard to children's family and educational backgrounds. During the course of 1 day, 89 German-speaking children participated in this study. The data of nine children had to be excluded from our analyses due to technical difficulties with the equipment (2), parental interference (1), because children had already done the experiment in a pretest-setting (2), because of probable cognitive impairments (1) or because of hit rates below chance in at least one paradigm (3). Since children were free to end their participation at any time, out of the remaining 80 children 58 completed all experimental paradigms and 22 completed one (10) or two (12) of the three tasks. During pre-processing of the data, we removed trials with more than one response and trials in which pictures were not shown correctly. Individual trials were excluded when RTs exceeded 4 s, were below 100 ms, or were more than 4 SDs from the child's mean RT.

In order to obtain meaningful results for comparing children's responses to the different paradigms, the main analyses presented here were conducted with the data of the 58 children who completed all three tasks. In order to take potential confounds of motivational and developmental differences into account, these results were then compared descriptively to those of the 22 children who completed a maximum of two experimental paradigms.

As mentioned above, children were subdivided a posteriori into three age groups to allow for a cross-sectional comparison. Detailed information on the number of children per age group, as well as age span, mean age and the amount of trials per age group and paradigm is provided in Table 1 for the main sample of 58 children.

**Table 1:** **Population characteristics and number of trials obtained per age group and paradigm**.

**Age group**	***N***	**Age (years)**		**% girls**	**Number of trials**		
		***M***	***SD***		**Human-bird-distinction**	**NoH-distinction**	**NoH-comparison**
Youngest (5–7 years)	14	6.07	0.83	37.93	851	862	451
Middle (8–9 years)	24	8.29	0.46	33.33	1552	1569	868
Oldest (10–13 years)	20	11.15	0.99	40.00	1286	1243	694
Total	58	8.74	2.11	37.93	3689	3674	2013

N, number of children; children's age was collected as age in years only. Human-bird-distinction, NoH-distinction, and NoH-comparison refer to the three different paradigms employed, see “experimental procedure” for details. Note that the maximum possible number of trials in NoH-comparison was n = 2436, and twice as many (n = 4872) in Human-bird-distinction and NoH-distinction.

### Stimuli

**Figure 1:**
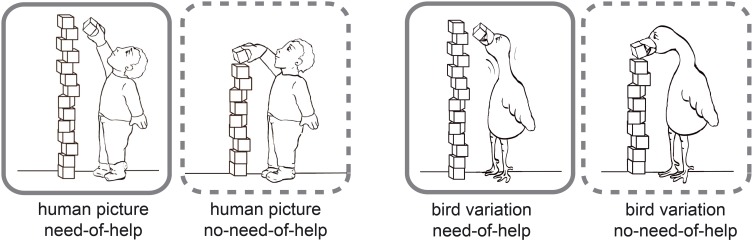
**Example stimuli**. Need-of-help (NoH) variations are framed by solid lines, no-need-of help (no-NoH) variations by dashed lines. A picture pair of a child (boy, kindergarten age) is shown on the left side, the corresponding bird picture pair on the right side.

The stimuli used are part of the NeoHelp stimulus set v01 (Brielmann and Stolarova, 2014) which comprises of 82 black-and-white comic drawings (for an example situation with its human and bird variations see Figure 1, all pictures used are presented in the Supplement S1). They show children of both genders and different ages in 15 everyday situations. The NeoHelp picture pairs for each of the 15 different everyday situations consist of one image depicting need-of-help (NoH) and a corresponding picture portraying no-need-of-help (no-NoH). As a control condition there are 15 stimuli pairs displaying birds in analog situations. The bird pictures serve to implement a control categorization task—Human-bird-distinction—and were created to ensure maximum perceptual similarity. In sum, there are 41 different pairs (NoH—no-NoH), 26 depicting children and 15 control picture pairs depicting birds in the same 15 situations as humans.

The stimuli were created as follows: A reference picture for each situation was drawn by hand and then converted into a black-and-white vector graphic using Adobe Illustrator CS 4. This format allowed us to create perceptually highly similar picture pairs and variations. Birds were chosen as control animal depictions since their body shape enabled us to maintain the highest perceptual similarity possible across both NoH/no-NoH and human/animal picture pairs. Within each NoH/no-NoH picture pair, differences only concerned those aspects of the picture that indicated NoH (e.g., child reaches for an apple, grasps it and thus succeeds in the attempt, or reaches for an apple, but it is too far away and the child's attempt fails). No-NoH depictions of picture variations were created by transferring changes in NoH-picture features made in the original to the no-NoH variation picture. Significantly, all conditions (NoH/no-NoH and human/bird) were created for each situation in order to ensure that the same stimulus material was usable across all three experimental paradigms. Detailed information about the stimulus set's properties as well its suitability for empirical investigations involving children have been provided by the authors in a separate methodologically oriented report (Brielmann and Stolarova, 2014).

### Setting and technical apparatus

The experiment took place at an open air child festival. Four trained experimenters were present at all times, both male and female, and assignment to computers and experimenters was random. One of the experimenters obtained informed written consent from the parents. The other three took care of the children at the computers, recorded the children's gender and age in years, as reported by the children themselves, as well as any peculiarities that occurred during testing. When children had completed one of the paradigms, they received a stamp. After completing all three paradigms, or when they lost interest, children received a small present. Depending mainly on the age of the children, total testing time per paradigm including instruction and training varied between 4 and 12 min.

Stimuli were presented on five laptop screens using the software Presentation (version 16.0). Five regular keyboards were adapted as response devices. All keys were covered with a cupboard contraption except for the two laminated and color-coded response keys. Color codes were counter-balanced for left and right responses across different PCs. Children were assigned to computers randomly. The laptop screens, as well as the table and the bench were kept in a constant place through the testing session. There were no constraints on the children's posture (e.g., no chin rest). Thus, the actual distance between a child's face and the screen varied between approximately 60 and 70 cm. Pictures measured between 15.62 and 11.75° of visual angle on the different PCs.

### Experimental design and procedure

**Figure 2:**
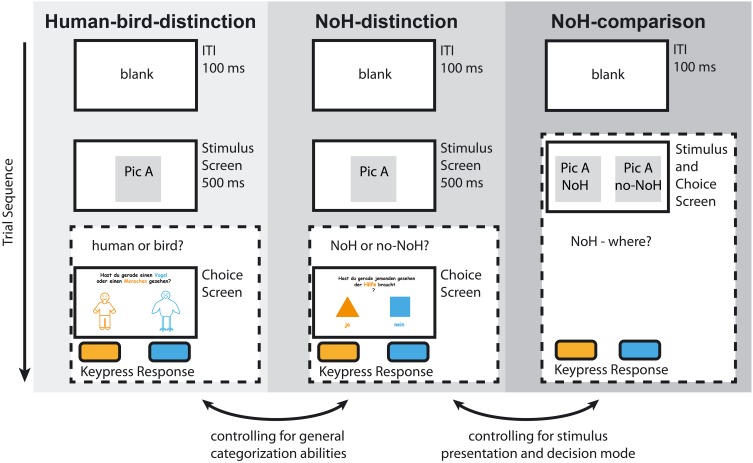
**Experimental paradigms**. The sequence for one example trial is illustrated for Human-bird-distinction (light gray), NoH-distinction (medium gray), and NoH-comparison (dark gray). Dashed frames indicate presentation without predefined time-restriction. Note that Human-bird-distinction and NoH-distinction had identical time restricted stimulus presentations (500 ms), randomization routines and experimental designs (2AFC); decision and response took place after stimulus off-set. Only the introduction and instruction differed between the Human-bird-distinction and the NoH-distinction paradigms. In the NoH-comparison paradigm stimuli were presented pair-wise and presentation was continued as long as needed for the child to respond. Responses were made during stimulus presentation. Labeled arrows at the bottom illustrate the mutual control between paradigms.

The experiment consisted of three research paradigms (see Figure 2) connected by a cover-story aimed at children (see below for details). Each paradigm started with a minimum of three training trials. The selection of the pictures for the training phase was random. All pictures (humans and birds, each with a need-of-help, and a no-need-of-help variation) were shown once in each paradigm so that differences in response characteristics cannot be attributed to differences in stimulus material. Inter-stimulus intervals were 100 ms long. Children were always instructed to respond as fast and as accurately as possible by pressing a designated button on their keyboard.

The first two paradigms had identical two-alternatives-forced-choice (2AFC) task setups. They differed solely in terms of instruction and thus task requirements. Stimuli were shown for 500 ms then a separate decision screen was presented. One 2AFC paradigm provided baseline measures for categorization abilities. It required children to decide whether they had just seen a human or a bird. This paradigm will subsequently be referred to as “Human-bird-distinction.” The second 2AFC paradigm asked children to indicate whether they had just seen someone (a human or a bird) in need of help (NoH) or not. It followed the same procedure as Human-bird-distinction and will be referred to as “NoH-distinction.” The third experimental paradigm also required need-of-help recognition, but a different task setup was employed in order to control for effects specific to the demanding 2AFC task. This NoH-distinction paradigm was a pair-wise picture-selection-task without time restrictions. It will be subsequently referred to as “NoH-comparison.” In NoH-comparison the corresponding NoH and no-NoH pictures (humans or birds) were presented side by side on the screen until the child made a response. Children were asked to indicate which out of two pictures within a pair depicted NoH. The order of the paradigms Human-bird-distinction and NoH-distinction was counterbalanced across different PCs. The assignment of children to PCs insured randomization. The NoH-comparison paradigm was conducted last, since the presentation and its duration made familiarization with the stimuli likely.

Each testing session was structured by a child directed cover story. Children were first greeted by an experimenter and introduced to Blobs, a friendly alien preparing to land on earth. Before starting the human-bird-distinction task, the experimenters explained that Blobs had lost his pet bird just before landing and needed help finding it. Therefore, the children needed to watch the flashing pictures carefully and indicate whether they could see a picture of a human or a bird (Blobs' lost pet). Before starting the NoH-distinction task, the experimenters explained that Blobs, while flying around Earth fast and catching only a glimpse of different situations, had difficulties understanding when someone on Earth needed help. Special care was taken to explain that it did not matter whether it was humans or birds who needed help, but that the general question was whether anyone needed help or not. So again, in order to help Blobs, children needed to watch the pictures flashing by very carefully and indicate after each one whether it depicted someone in need of help or not. In the NoH-comparison task, the experimenter explained to the children that Blobs was still confused about behavior on Earth and continued to have difficulties understanding if and when somebody needed help. Children had to indicate whether the help-variation was shown on the left or the right side. After each paradigm, children saw a happy and grateful Blobs and were praised for helping him.

Paradigms were designed to explore effects specific to the need-of-help recognition process: Comparing results of the two 2AFC tasks NoH-distinction and Human-bird-distinction disentangled influences of basic categorization abilities (and thus of general developmental characteristics such as speed of processing) from need-of-help recognition abilities. The comparison of results for NoH-distinction and NoH-comparison tasks disentangled influences of task load through time restriction from those of NoH recognition. The visual stimuli were kept constant across the three experimental paradigms. Thus, any effect that emerges in both help-related paradigms (NoH-distinction and NoH-comparison) and not in the Human-bird-distinction task should be attributable to the need-of-help recognition abilities of children. Effects that remain similar across the two 2FAC tasks are most probably attributable to general categorization abilities and speed of processing.

### Analysis strategy

All our statistical analyses were conducted using GNU-software R (version 3.0.0 and 3.0.2). The analyses concerning response characteristics were conducted twice: once for hit rates and once for RTs. As results comparing effects and response characteristics across paradigms are only meaningful if obtained from the same population, all analyses were conducted with the data of children who had completed all paradigms (*N* = 58). The subsample of children not completing all paradigms (*N* = 22) was only considered to descriptively compare results (see below).

In an exploratory analysis we first assessed how response characteristics were influenced by age and gender by means of 3 × 2 (*age group* × *gender*) ANOVAs for each paradigm as omnibus tests. As data of each child was thus analyzed three times, the significance level was conservatively adjusted to *p* < 0.017. Scheffé-tests were used to investigate *post-hoc* group differences. In order to assess whether exclusion of children not completing all paradigms contributed to the effects observed, we also calculated the effect sizes for gender and age group differences in the sample of children not completing all paradigms (*N* = 22). Meaningful analog ANOVAs could not be calculated for this subsample as there were empty cells for the combination of age group and gender in each paradigm when considering this sample. Cohen's d served as an estimate of effect sizes applicable to both samples.

Secondly, we employed correlation analyses in order to test whether and how children's responses were associated across experimental paradigms using Pearson's correlations. We also tested whether the hit rates for human- and bird-depictions correlated. Correlations were analyzed for boys (*N* = 36) and girls (*N* = 22) separately. Thirdly, we investigated whether gender differences were explicable by own-gender effects depending on the task using a 3 × 2 × 2 (*paradigm* × *gender* × *picture-gender*) ANOVA.

## Results

### Population characteristics

The age of the 58 children who completed all three paradigms ranged from 5 to 13. Children were then further subdivided into three age groups (see Table 1). Age distribution did not deviate from equal distribution, *p* = 0.09. Across age groups no systematic differences in gender distribution were found, χ^2^_(1, *N* = 58)_ = 0.40, *p* = 0.82. The absolute numbers of trials obtained for each paradigm in each age group are shown in Table 1. Children who did not complete all paradigms (*N* = 22) showed similar demographic characteristics: A Kolmogorov-Smirnov test revealed non-different age distributions, *D* = 0.53, *p* = 0.19 and Chi-squared tests showed that gender proportions were not different between the groups, χ^2^_(2, *N* = 58)_ = 0.02, *p* = 0.90.

### Influences of age and gender on hit rates in need-of-help recognition

**Figure 3:**
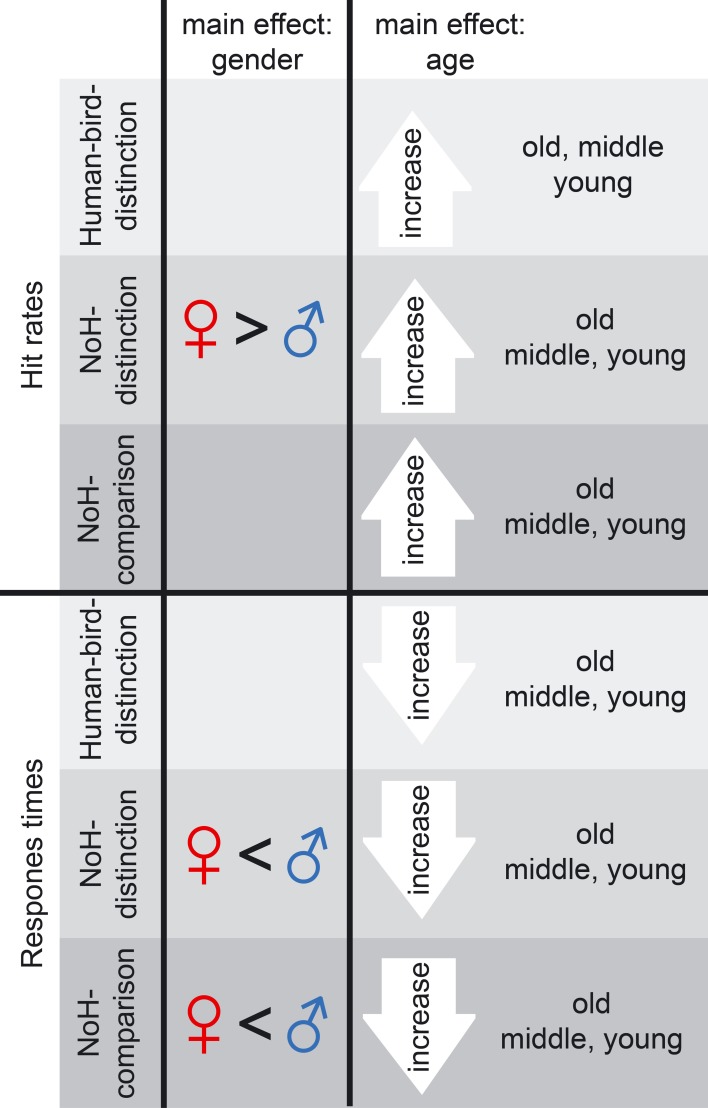
**Summary of age and gender main effects**. Main effects on hit rates (top) as well as on RTs (bottom) are shown for gender (left) and age (right). Main effects are shown for Human-bird-distinction (light gray), NoH-distinction (medium gray) and NoH-comparison (dark gray). Note that main effects of gender were present in help-related paradigms only.

Main effects of *age group* and *gender* on hit rates are illustrated in the top half of Figure 3. Effect sizes are given as Cohen's d for effects of gender as well as age in the columns 3–6 of Table 2.

**Table 2:** **Effect sizes for gender × age group interaction effects for the three different paradigms**.

**Paradigm**	**Age group**	**Hit rates**			**RTs**		
		***d* (gender)**	***d* (age) girls**	***d* (age) boys**	***d* (gender)**	***d* (age) girls**	***d* (age) boys**
Human-bird-distinction	Youngest	−0.05	**−0.33**^a^	−0.12^a^	**−0.47**	**−0.43**^a^	**0.21**^a^
	Middle	0.03	−0.03^a^	−0.13^a^	0.08	**0.64**^a^	**0.38**^a^
	Oldest	−0.03	**−0.35**^b^	**−0.26**^b^	−0.05	0.18^b^	**0.61**^b^
NoH-distinction	Youngest	**0.23**	0.09^a^	−0.07^a^	**−0.54**	−0.17^a^	0.05^a^
	Middle	0.13	−0.12^a^	**−0.20**^a^	**−0.31**	**0.23**^a^	**0.43**^a^
	Oldest	0.01	−0.03^b^	**−0.27**^b^	−0.01	0.05^b^	**0.48**^b^
NoH-comparison	Youngest	**0.40**	0.18^a^	**−0.23**^a^	**−0.46**	−0.05^a^	**0.28**^a^
	Middle	0.13	−0.03^a^	−0.15^a^	**−0.29**	**0.26**^a^	**0.50**^a^
	Oldest	−0.04	0.16^b^	**−0.39**^b^	−0.07	**0.20**^b^	**0.80**^b^

a Comparing row's mean to oldest age group.

b Comparing row's mean to youngest age group.

Youngest = 5–7 years; Middle = 8–9 years; Oldest = 10–13 years. Numbers in bold indicate small (d ≥ 0.2), medium (d ≥ 0.5), or large (d ≥ 0.8) effect sizes.

**Figure 4:**
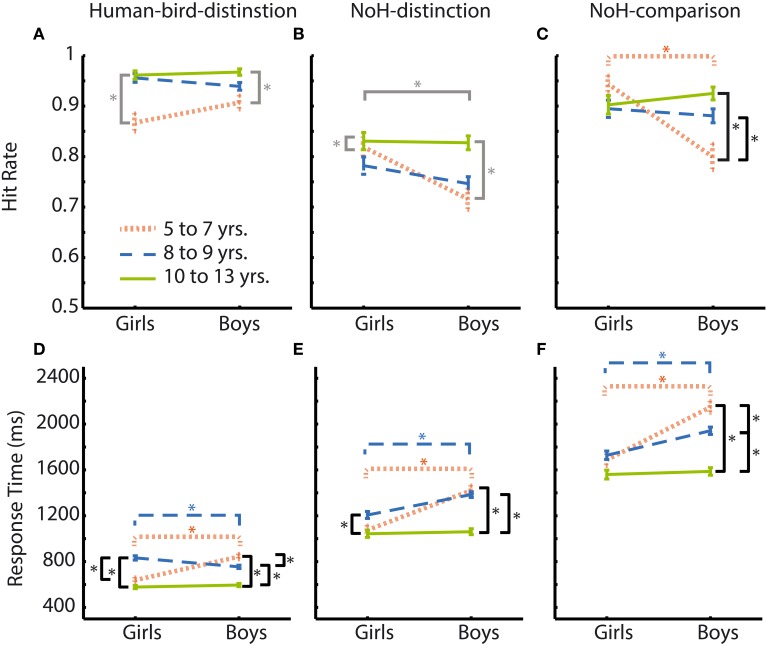
**Hit rates and RTs according to age group and gender for all paradigms**. Age groups are represented by different colors: youngest = orange thinly dashed lines, middle = blue broadly dashed lines, oldest = green solid lines. Girls' responses are shown on the left side of each graph, boys' responses on the right. Comparisons between age groups and genders are illustrated for Human-bird-distinction (left, **A,D**), NoH-distinction (middle, **B,E**) and NoH-comparison (right, **C,F**). Differences between age groups when considering the interaction *age group* × *gender* are indicated in black. Significant differences between boys and girls are marked according to age groups' colors. Differences resulting from main effects not accompanied by an interaction are marked in gray. Asterisks mark significant differences according to *post-hoc* Scheffé-tests. Error bars represent SEM.

Hit rates were generally high across experimental paradigms and age groups for boys as well as girls with mean hit rates ranging between 0.97 and 0.72. In the Human-bird-distinction task, in which need-of-help content was irrelevant, no effects of *gender* were found, main effect: *F*_(1, 3683)_ = 0.0.38, *p* = 0.54; interaction: *F*_(2, 3683)_ = 3.76, *p* = 0.02. At the same time, a main effect of *age group* emerged, *F*_(2, 3683)_ = 26.02, *p* < 0.001. *Post-hoc* Scheffé-tests showed that the oldest children had higher hit rates than both younger age groups (see Figure 4A; Table 2).

In contrast to Human-bird-distinction, *gender* had a main effect on hit rates in NoH-distinction, *F*_(2, 3668)_ = 8.84, *p* < 0.01, showing that girls generally had higher hit rates in this demanding (short presentation times and decision from memory) need-of-help recognition task (see Figure 4B). *Age group* had a similar effect on hit rates in NoH-distinction as in Human-bird-distinction, *F*_(2, 3668)_ = 11.12, *p* < 0.001, with *post-hoc* comparisons showing that the oldest children had higher hit rates than the two younger age groups. These effects were not further moderated by an interaction, *F*_(1, 3668)_ = 3.74, *p* = 0.02. Note that the magnitude of differences between boys and girls with regard to hit rates decreased with age in this need-of-help recognition paradigm (see column 3 of Table 2).

For the NoH-comparison task we did not find a main effect of *gender* on hit rates, *F*_(1, 2007)_ = 5.08, *p* = 0.02. The main effect of *age group* was significant, *F*_(2, 2007)_ = 4.10, *p* < 0.017. However, *age group* and *gender* interacted, *F*_(2, 2007)_ = 10.26, *p* < 0.001. In fact, boys and girls only differed with regard to hit rates in the youngest age group (see orange line Figure 4C). Moreover, age differences were only observed for boys: The youngest boys showed lower hit rates compared to those who were older. Interestingly, the youngest and mid-range aged girls did not perform differently from the oldest boys and girls. The youngest girls (aged 5–7) showed higher hit rates in need-of-help recognition than boys of the same age when no-NoH and NoH pictures were shown side by side and reached a performance level similar to the oldest age group. As in the NoH-distinction task, the magnitude of differences between boys and girls decreased with age (see column 3 of Table 2). The relative advantage of the youngest girls makes their performance comparable to that of the oldest girls and boys and contributes to the finding that gradual age-related accuracy increases were only present for boys (see column 5 of Table 2; Figure 4C).

In summary, the above results provide evidence that the gender-related differences in accuracy found for the younger children are specific to need-of-help recognition. Younger girls show higher hit rates in need-of-help recognition than boys of the same age. These differences between boys and girls decreased with age and were not present over 9 years of age (see column 3 of Table 2). As these gender differences did not emerge in the Human-bird-distinction task at all, they are unlikely to reflect a general developmental or motivational advantage. Girls' accuracy across all three age groups in a non-time restricted comparison task did not differ systematically. Boys on the other hand showed the expected age related gradual accuracy increase.

### Influences of age and gender on RTs in need-of-help recognition

RTs were more sensitive to influences of *age group* and *gender* as well as to their interactions than hit rates. Main effects of *age group* and *gender* on RTs are illustrated in the bottom panel of Figure 3. Effect sizes are given as Cohen's d for effects of *gender* as well as *age* in the three rightmost columns of Table 2. Similarly to what results have shown for hit rates, *gender* had less consistent and less strong effects on RTs in the not help-content related Human-bird-distinction task (compare topmost three rows to the rest of Table 2). The main effect of *gender* was not significant, *F*_(1, 3683)_ = 2.59, *p* = 0.11. Nonetheless, gender interacted with *age group*, *F*_(2, 3683)_ = 30.18, *p* < 0.001, which was not the case for accuracy raters in this paradigm. At the same time, *age group* also had a main effect on RTs, *F*_(2, 3683)_ = 83.54, *p* < 0.001. The pattern of gender differences in this paradigm was as follows (see Figure 4D): In the younger age categories girls responded faster than boys but this was no longer the case in the oldest group. RTs decreased steadily for boys as they got older. For girls, RT patterns were less clear and did not differ significantly for younger or older girls: Girls aged 8 and 9 showed RTs that were on average longer than those of both younger and older girls, and also lower than boys of the same age (see Figure 4D).

In the first help-content related NoH-distinction task, all effects were significant, *F*_(2, 3668)_ = 44.09, η^2^_*p*_ = 0.02, *F*_(1, 3668)_ = 42.80, and *F*_(2, 3668)_ = 12.11, for main effects of *age group*, *gender* and their interaction respectively, all *p* < 0.001. *Post-hoc* tests revealed significant differences between boys and girls in the youngest and middle age group, but not in the oldest one (see Figure 4E). This gender effect was stronger for the youngest compared to the middle age group (Table 2). The oldest boys reacted fastest compared to both younger age groups. Also, the oldest girls reacted faster than girls from the middle age group.

In NoH-comparison, RTs showed the same pattern of effects as observed in the NoH-distinction task (compare Figures 4E,F): Both main effects and the interaction of *age group* and *gender* were significant, *F*_(2, 2007)_ = 48.57, *F*_(1, 2007)_ = 41.90 and *F*_(2, 2007)_ = 12.78, respectively, all *p* < 0.001. Mirroring findings from all three paradigms, *post-hoc* tests revealed that girls responded faster than boys in the youngest and middle age group, but not in the oldest age group. This gender effect was moderate for the youngest age group and only small for the middle age group (Table 2). For boys RTs decreased steadily with age. In contrast, there was no age-related change in RTs for girls. Remarkably, there were no systematic differences in RTs for the youngest girls compared to the oldest children in this non-time restricted help-content related task as the (see Figure 4F).

In summary, the impact of age on RTs was similar across the three experimental paradigms and thus cannot be attributed solely to differences in the ease and speed of processing of need-of- help related content. The interaction of *age group* and *gender* revealed highly similar patterns across all three experimental paradigms, too. Pronounced differences between boys and girls were visible across the three tasks only in the youngest age group. The magnitude of gender effects in the middle age group reached a meaningful level only in need-of-help recognition tasks, no differences between boys and girls regarding RTs were observed for children between 10 and 13 years (see column 6 of Table 2).

### Stability of age and gender effects across subsamples

In order to assess whether the results reported above are restricted to particularly motivated or more developmentally advanced children we also analyzed the subsample of 22 children who had decided to end their participation before completing all three paradigms. Because of the small sample size resulting in empty cells, a formal statistical comparison of the two subsamples was not possible. We will thus provide a descriptive comparison and report effect sizes where possible.

**Figure 5:**
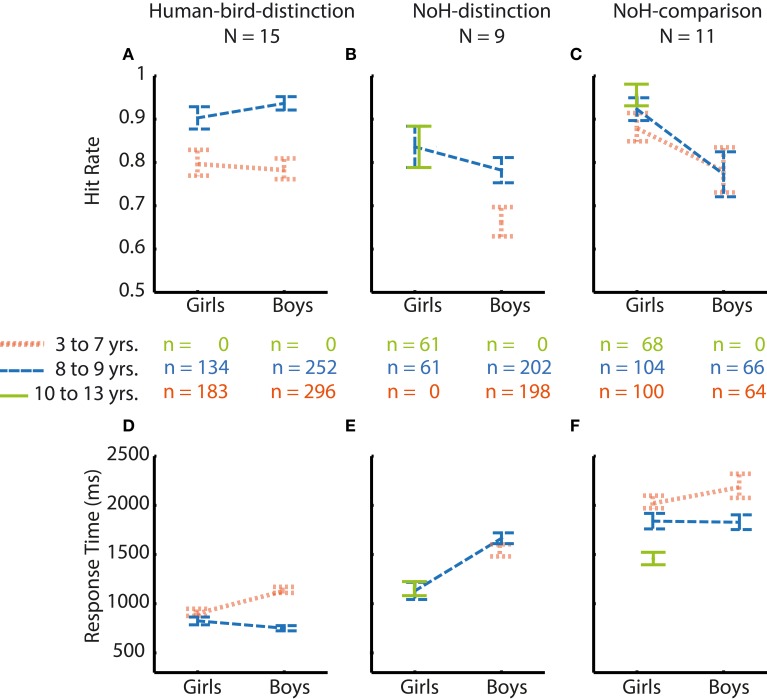
**Hit rates and RTs for those children only completing one or two paradigms**. Data is shown according to *age group* (youngest = orange thinly dashed lines, middle = blue broadly dashed lines, oldest = green solid lines) and *gender* for all paradigms. Girls' responses are shown on the left side of each graph, boys' responses on the right. Comparisons between age groups and genders are illustrated for Human-bird-distinction (left, **A,D**), NoH-distinction (middle, **B,E**) and NoH-comparison (right, **C,F**) separately. N refers to the number of children, n to the number of trials. Error bars represent SEM.

Comparisons of response patterns across age groups and genders of those 22 children who did not absolve all paradigms were similar to those obtained in our main sample (compare Figures 4, 5). This was also evident when considering the magnitude of differences between boys and girls in this smaller subsample (compare Tables 2, 3): Systematic differences in accuracy emerged only in the NoH-comparison paradigm. Girls tended to have higher response accuracies in the two need-of-help related tasks only. Differences between age groups, if assessable, were larger for RTs compared to hit rates. Age differences were larger for the Human-bird-distinction paradigm compared to the help-related paradigms. Thus, even though a formal comparison across the two subsamples of children was not possible, the obtainable result patterns were similar for both subsamples. Therefore, the reported pattern of results is unlikely to be restricted to highly motivated or unusually advanced children, but seems rather stable.

**Table 3:** **Effect sizes for gender and age group differences for the three different paradigms in the subsample of children not completing all paradigms (*N* = 22)**.

**Paradigm**	**Age group**	**Hit rates**			**RTs**		
		***d* (gender)**	***d* (age) girls**	***d* (age) boys**	***d* (gender)**	***d* (age) girls**	***d* (age) boys**
Human-bird-distinction	Youngest	0.03	**−0.29**^a^	**−0.44**^a^	**−0.45**	0.17^a^	**0.79**^a^
	Middle	−0.13	−	−	0.17	−	−
	Oldest	−	−	−	−	−	−
NoH-distinction	Youngest	−	−	**−0.27**^a^	−	−	−0.16^a^
	Middle	0.13	0^a^	−	**−0.71**	−0.04^a^	−
	Oldest	−	−	−	−	−	−
NoH-comparison	Youngest	**0.27**	−0.14^a^	0.02^a^	**−0.21**	**0.26**^a^	**0.45**^a^
	Middle	**0.45**	−0.13^a^	−	0.01	**0.52**^a^	−
	Oldest	−	**−0.27**^b^	−	−	**0.94**^b^	−

a Comparing row's mean to oldest age group.

b Comparing row's mean to youngest age group; dashes indicate that there was no data for at least one comparison group.

Youngest = 3–7 years; Middle = 8–9 years; Oldest = 10–13 years. Numbers in bold indicate small (d ≥ 0.2), medium (d ≥ 0.5), or large (d ≥ 0.8) effect sizes.

### Correlation analyses of response characteristics

**Figure 6:**
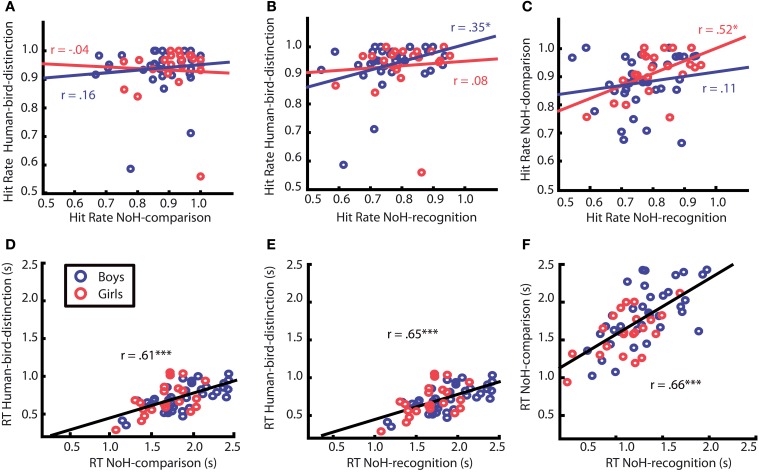
**Correlation of hit rates (top) and RTs (bottom) between different paradigms**. Each data point corresponds to the mean values for one child. Red circles represent girls, blue ones boys. Correlation patterns differed between girls and boys only for hit rates (see upper row **A**, **B** and **C**), but did not for response times (RTs, see bottom row, **D**, **E** and **F**). Red and blue regression lines and adjacent correlation coefficients refer respectively to data of girls and boys. Black regression lines and correlation coefficients, for which emergence of correlations did not differ for girls and boys, refer to data of all children. Asterisks mark significant Pearson correlation coefficients, ^*^p < 0.05; ^***^p < 0.001. Regression lines were created using MatLab LSD.

Correlation analyses were conducted to detect systematic relations between children's responses across experimental paradigms. Correlations of hit rates across paradigms were calculated separately for boys and girls. For girls hit rates in the two help-related tasks (NoH-distinction and NoH-comparison) correlated significantly (see Figure 6C), *p* = 0.01. This was not the case for boys, *p* = 0.34. For them, hit rates in the two paradigms with the same (fast) picture presentation mode, but with different task content (Human-bird- and NoH-distinction) were positively linked (see Figure 6B), *p* = 0.04. This was not the case for girls, *p* = 0.74.

**Figure 7:**
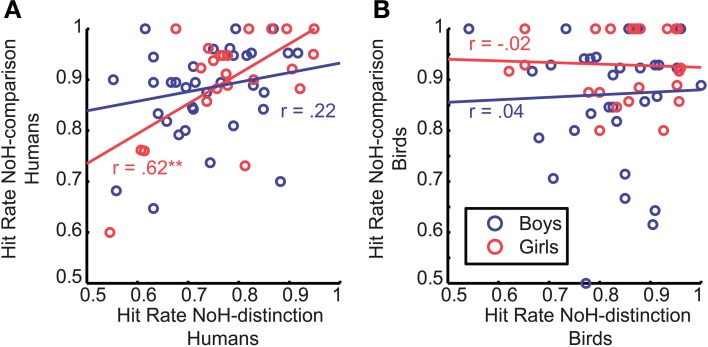
**Correlation of hit rates between the two need-of-help recognition paradigms**. Data is shown separately for human- **(A)** and bird-depictions **(B)**. Each data point corresponds to the mean values for one child. Red circles refer to girls, blue ones to boys. Red and blue regression lines and adjacent correlation coefficients refer respectively to data of girls and boys. The asterisk indicates the significant Pearson correlation; ^**^p < 0.01. Regression lines were created using MatLab LSD.

We were able to further specify what is different in need-of-help recognition for girls compared to boys by splitting data into two subgroups: pictures of humans and pictures of birds. It turned out that the correlation between hit rates of the two help-related tasks (NoH-distinction and NoH-comparison) described for girls is only present when pictures of humans are shown, *p* < 0.01 for humans; *p* = 0.93 for birds. For boys, the hit rates of NoH-distinction and NoH-comparison were uncorrelated for depictions of both humans and birds (see Figure 7), both *p* = 0.19.

The mean values for RTs across all paradigms correlated highly with each other, all *r* = 0.61, all *p* < 0.001. The bottom row of Figure 6 shows the corresponding scatter plots including correlation coefficients. The strength of correlations did not differ between paradigms, *z* = 0.46. Thus, children who responded faster did so in all paradigms whatever the demands of the task. These strong associations generalized for human and for bird depictions, for boys as well as for girls. The correlation analyses provided additional evidence that the abilities captured by children's RTs were not specific to need-of-help content (as was the case for response accuracies) and its ease of processing, but rather reflected more general abilities of the children, e.g., speed of processing.

### Relation between observers' and picture's gender

Only responses to depictions of humans with an identifiable gender (e.g., not pictures showing babies or toddlers) were included in this analysis (*n* = 3996). The gender of the depiction did not influence neither hit rates nor RTs, both *F*_(1, 3984)_ = 1.63, both *p* = 0.25, both η^2^_*p*_ = 0.00, and did not interact with neither children's *gender* or *paradigm*, all *F*_(2, 3984)_ = 1.82, all *p* = 0.16, all η^2^_*p*_ = 0.00.

## Discussion

The capacity to identify a situation in which someone needs help is a necessary precondition for initiating helping behavior. It is therefore an important aspect of children's social development. The present study assesses whether previously reported age and gender differences with regard to some aspects of active helping and social perception are also evident in a child's ability to recognize another person's need-of-help. In this way, we separate need-of-help recognition as an early socio-perceptual process from later processing stages leading to active helping, such as motivation and choosing to help. In a computer-based exploratory study, children were asked to indicate whether someone (a human child or a bird) shown on a picture was in need of help. We measured two behavioral correlates: response accuracy and RT. Both have been shown to develop throughout childhood in a variety of tasks, including differentiation of emotional stimuli (e.g., Kail, 1993; Gao and Maurer, 2009; Kail et al., 2013; for an overview of developmental changes in cognitive control see Davidson et al., 2006). Therefore, a control task was used to provide baseline measures for these response characteristics independently of need-of-help recognition. This control task required children to indicate whether they had seen a human or bird. Importantly, all three experimental paradigms employed the identical visual stimuli in order to ensure that variations in response patterns were attributable to differences in information processing and decision characteristics only—not to differences in stimulus material. Below we first discuss response accuracy as a measure of need-of-help recognition capacity before turning to children's RTs which are assessed here as an indication of ease of processing and decision.

Overall, high accuracies were observed across all experimental paradigms, providing evidence that the tasks were doable for children in all three age groups: They reliably recognized situations in which a human child or a bird was in need of help and distinguished between humans and birds. Even the somewhat abstract and unrealistic depictions of birds in human-like need-of-help situations did not pose difficulties to this normative population of children aged 5–13 years when it came to recognizing need-of-help content.

The children's need-of-help recognition accuracy increased with age, as would be expected for a variety of differentiation tasks (see for example Davidson et al., 2006). This was, however, not equally true for boys and girls. Moreover, age-related increases in hit rates differed systematically between need-of-help related tasks and the Human-bird-distinction task. In both need-of-help recognition tasks, but not in the control Human-bird-distinction task, a clearly higher accuracy of girls' responses compared to boys' responses was evident for the youngest age group (5–7 years, see Figures 4B,C). In the (less demanding) NoH-comparison task girls of all three age groups even recognized the need-of-help at a performance level that did not differ statistically from that of the oldest children (see Figure 4C). This means that the 5–7 years old and 8–9 years old girls exhibited average need-of-help recognition accuracies similar to those of boys and girls aged 10–13. Boys showed the expected gradual improvement of accuracy in both need-of-help recognition tasks as well as in the control Human-bird-distinction task. Girls' accuracy, on the other hand, was noticeably high and stable across age groups in the need-of-help related tasks. Our results thus show that while boys demonstrate a gradual accuracy increase with age that contributes to the absence of any gender differences in the oldest age group (here 10–13 years of age), girls' need-of-help recognition abilities tend to be high from an early age on. Differences between boys and girls in hit rates were restricted to the two need-of-help related tasks, but were not at all present in the Human-bird distinction task. In this control task a gender independent improvement of performance with age was observed, as would be expected for categorization tasks relying on perceptual abilities (Kail, 1993; Batty and Taylor, 2002). Thus, the observed accuracy advantage of the younger girls is likely to be specific to need-of-help recognition abilities, not attributable to general developmental or motivational differences between boys and girls. This conclusion was further substantiated by the fact that a smaller subsample of children unable or unwilling to complete all three tasks exhibited response patterns similar to those described above.

Since this is the first study to investigate children's need-of-help recognition capacity, we cannot directly relate the present findings to previous literature. The data discussed here is not sufficient to identify the underlying mechanisms or aspects of the processes leading to systematic age-specific differences between boys and girls. The observed differences between younger boys and girls might, however, be explained within the larger framework of differences between men and women in processing of socially relevant content. This has already been discussed with regard to socially relevant stimuli, such as biological motion or social scenes (Proverbio et al., 2008: Anderson et al., 2013) and processing of purposeful actions in particular (Proverbio et al., 2010). It could be that girls allocate a higher amount of attention to the socially relevant content of the presented visual stimuli from an early age on, while for boys the relevance of the stimuli' social content increases gradually with age. Whether this is a valid explanation cannot be resolved completely on the ground of the present data, but systematic associations between response accuracies for different tasks provide helpful indications (see Figures 6A–C). We found that girls' accuracy correlated across need-of-help related tasks. Boys' response accuracies did not correlate across the two help content related tasks, but were related across both paradigms with identical time-restricted presentation and decision after stimulus offset regardless of the different content of the two tasks (NoH- vs. Human-bird-distinction, Figure 6B). This means that girls who showed high accuracy in one of the two no-need-of-help related tasks also tended to do so in the other one. In contrast, boys accuracies correlated under the condition of short presentation time and decision from memory regardless of task content (see Figure 6). Thus, we observed systematic differences regarding which features of a task elicited coherent behavioral responses in boys and girls. This finding could indicate that girls allocate more attention or other information processing resources to socially relevant picture content than boys. This observation is further differentiated by the fact that the correlation between accuracies in the two help-related paradigms observed for girls was carried by their responses to pictures of humans (compare Figures 7A,B). We did not find such a human-specific accuracy correlation for boys. We propose that the importance of the pictures' socially relevant content might have been greater for girls than for boys. Further studies are necessary to test these assumptions. Systematic measures of attention allocation and its psychophysiological correlates would help determine whether gender related performance differences reported here reflect systematic differences in early information processing. Also, future studies including infants and younger children which take a broader range of social, cultural and biological influences into account will help to clarify to what extend the gender related differences in this study are the result of an interaction between biological and socio-cultural influences. At this point we can only assume that the reported findings cannot solely originate from either biological or social factors.

Generally, the magnitude of differences between boys and girls in need-of-help recognition tasks decreased with age. No differences were detectable between boys and girls in the oldest age group (10–13 years); they were moderate in the middle age group (8–9 years) and largest in the youngest age group (5–7 years, see Table 2). These results appear to contrast with the finding that gender differences in neuronal correlates of social perception increase with age (Anderson et al., 2013). However, it must be pointed out that the participants in Anderson's study only viewed socially relevant stimuli (biological motion) and non-social stimuli (scrambled motion) passively. It might thus be that for decision processes, as assessed in our study, differences between boys and girls follow a different developmental trajectory compared to socio-perceptual processes alone. It might also be that the gender differences described with regard to children's brain activity while passively viewing visual stimuli (Anderson et al., 2013) are already sufficient for generating differences between boys and girls in our tasks. An additional explanation for diminishing gender differences with age could also be sought in the subjectively perceived difficulty of the tasks: The high and stable performance levels of the oldest children suggest a ceiling effect, i.e., the employed paradigms might have been too simple to elicit any meaningful differences in performance accuracy of the older children. Indications for increasing ease of processing with age is also provided by the fact that children's RTs decreased with age across tasks.

Measures of response accuracy and measures of speed of response are sensitive to different aspects of processing in adults (Santee and Egeth, 1982; Prinzmetal et al., 2005), as well as in children (Davidson et al., 2006). Mirroring these broadly documented findings, children's RTs in this study tell a somewhat different story than their performance accuracies. We found the expected age related decrease in RTs (e.g., Ratcliff et al., 2012; Kail et al., 2013) across all three experimental paradigms (see Figures 4D–F). As opposed to findings regarding children's accuracy, differences between boys and girls in RTs were present for the youngest and middle age groups across all three experimental paradigms. For the oldest boys and girls (10–13 years) no gender differences in RTs were observed in any of the three experimental paradigms just as there were no such differences in accuracy. The magnitudes of gender related differences for the two younger age groups were small to moderate (see Table 2). These results rule out a possible explanation that the youngest girls have an advantage in need-of-help recognition accuracies by means of a speed-accuracy tradeoff. They also show that (younger) girls are not specifically faster at recognizing need-of-help content but rather respond faster compared to same age boys across all three experimental paradigms employed here. Still, only in need-of-help recognition tasks, differences between boys and girls were also evident in the middle age group, albeit moderate in size (see Table 2). This pattern of results could be seen as an indication that an initially generally faster response speed of younger girls turns into a more specific need-of-help related advantage in the middle age group and diminishes completely for the oldest children. Further research is necessary to determine cause and extend of faster RTs for younger girls and the reasons it diminishes with age. In light of previous research on social perception (e.g., Proverbio et al., 2008; Anderson et al., 2013) it would be interesting to find out whether younger girls would also respond faster to socially irrelevant stimuli. On the basis of the data available, we cannot determine whether the observed faster RTs of younger girls reflect a general developmental advantage or whether they are restricted to pictures of humans and birds (or other animals). Such pictures of animate objects might carry higher social significance than pictures of objects or scenes as e.g., used by Proverbio et al. (2008).

In light of previous results showing own-gender related associations between the actor and observer's gender in social perception (Kret and De Gelder, 2012) and differences in prosocial behavior (e.g., Eagly, 2009), we investigated whether the children in our study might demonstrate specific response patterns depending on the gender of the depicted person. We found no indication for such own-gender effects when merely a decision regarding need-of-help was required: No interaction of the pictures' and children's gender was observed in either response accuracy or RTs. Thus, differences between boys and girls in our study cannot be explained by means of greater reactivity or expertise to own-gender depictions. Moreover, these results show that the gender of the depicted child, a task-irrelevant aspect of picture content, had no influence on response characteristics in this study. Whether different kinds of stimuli, such as for example photographs or portraits containing clearer references to a person's gender than the comic-like drawings employed here, are more likely to induce own-gender effects, remains to be determined in future research.

In conclusion, our study shows that need-of-help recognition abilities in a normative child population improve with age; developmental trajectories differ for boys and girls. We found a relative advantage of 5–7 years old girls compared to boys of the same age specifically regarding accuracy of need-of-help recognition. We also found systematic tendencies for faster RTs of younger girls that are not restricted to need-of-help recognition, but seem to be of more general nature. The magnitude of all reported gender effects decreased with age, no indication for systematic differences in either hit rates or RTs between boys and girls above the age of 9 years was found in any experimental paradigms. One possible explanation for the higher response accuracy of younger girls could be that the social information was more relevant for girls than for boys in the present context, as indicated by correlation findings. The present study does not provide any indication, whether the observed gender related differences result in motivational differences between boys and girls (see Hepach et al., 2013a, for a discussion of intrinsic vs. extrinsic motivation for prosocial behavior) or in differences with regard to quality or quantity of active helping. It shows, however, that the initial accuracy of need-of-help recognition differs between boys and girls at younger ages, and that gender related differences diminish with increasing age. These results can serve to explain some of the gender related variance in prosocial behavior. They put forward the need to not only assess the motivation to help or the performance of helping behavior, but also its perceptual precedents. Only then those different stages of helping can be linked together. Moreover, our results add evidence to the notion that social stimuli, especially such involving purposeful human action, might be treated with higher priority by female participants already in early childhood. Further studies are needed to uncover the mechanisms underlying need-of-help recognition and the observed gender differences in early childhood, as well as to determine which factors drive these differences in younger children and which factors in turn lead to an extinction of these effects in older age groups.

### Conflict of interest statement

The authors declare that the research was conducted in the absence of any commercial or financial relationships that could be construed as a potential conflict of interest.

## Footnotes

^1^We use the term “gender” rather than “sex” deliberately here, since it describes the interactive influence of a larger variety of social and biological factors, while the term “sex” refers to biologically determined differences between males and females (for discussion see e.g., Unger and Crawford, 1993; Torgrimson and Minson, 2005).
