# Supplementary material for: Does anyone need help? Age and gender effects on children's ability to recognize need-of-help
Source: Front Psychol. 2014 Feb 27;5:170. doi: 10.3389/fpsyg.2014.00170 (PMC3936112; doi:10.3389/fpsyg.2014.00170)

**Visual stimuli employed in this study**

A total of 82 different visual stimuli was employed; all of them were part of the NeoHelp Stimulus Set[1]. Stimuli were created for 15 different everyday situations.

| | | | | | |
|---|---|---|---|---|---|
| 1 | Apple | 6 variations | 9 | Gap | 6 variations |
| 2 | Blocks | 6 variations | 10 | Shelf | 6 variations |
| 3 | Boat | 6 variations | 11 | Shirt | 4 variations |
| 4 | Branch | 6 variations | 12 | Sit | 4 variations |
| 5 | Bucket | 6 variations | 13 | Stair | 6 variations |
| 6 | Climb | 4 variations | 14 | Table | 4 variations |
| 7 | Door | 4 variations | 15 | Table_chair | 8 variations |
| 8 | drawer | 6 variations | | | |

Pictures were created pairwise: one image depicts a child in need-of-help (NoH), the corresponding picture portrays no-need-of-help (no-NoH) while changing picture content as little as possible. As a control condition there are 15 stimuli pairs displaying birds in analog situations. Also, variations of child-depictions were created to increase the number of stimuli. Special care was taken that differences between pictures are restricted to changes in as little features as possible by successively deriving pictures from one first reference picture:

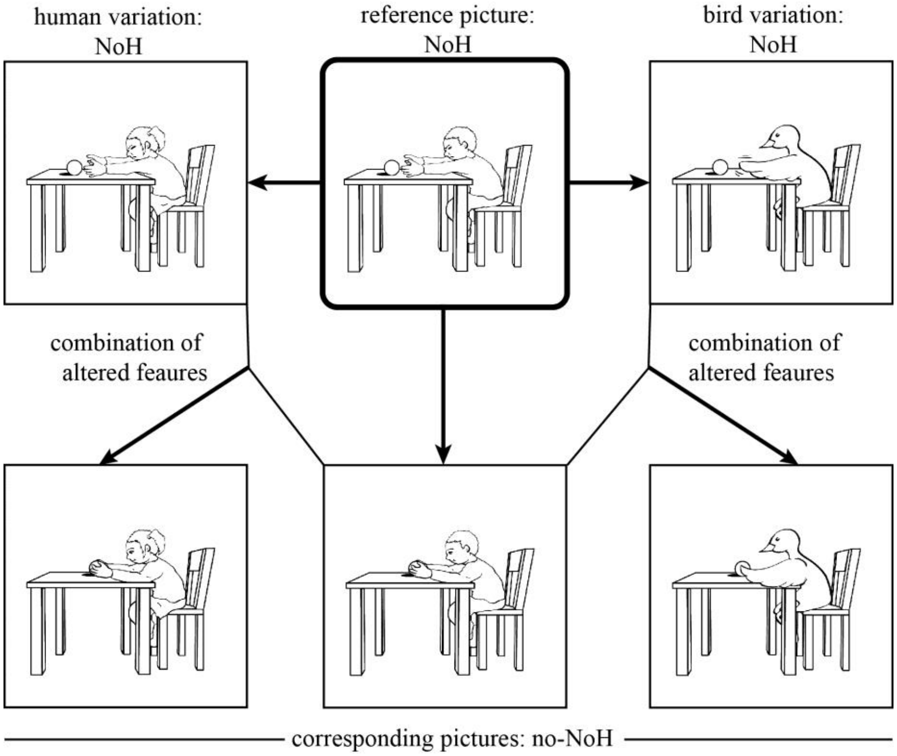


The following pages list all pictures of the NeoHelp stimulus set v01 in this alphabetical order. Corresponding need-of-help (NoH) and no-need-of-help (no-NoH) depictions are shown directly one above the other.

[1] Detailed information about the stimuli's characteristics is accessible here: Brielmann, A. A., & Stolarova, M. (2014). A New Standardized Stimulus Set for Studying Need-of-Help Recognition (NeoHelp). *PLOS ONE*, *9*(1), e84373.

“apple”

apple
NoH

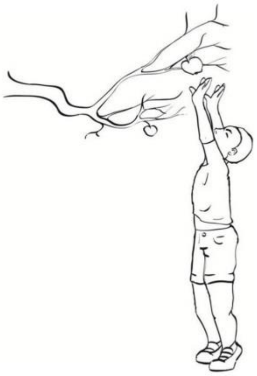

apple
girl
NoH

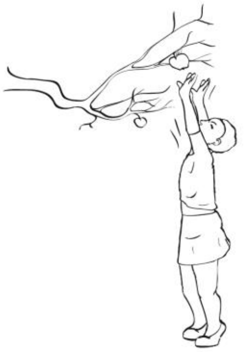

apple
no-NoH

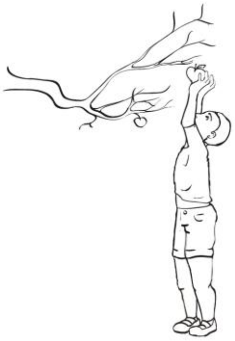

apple
girl
no-NoH

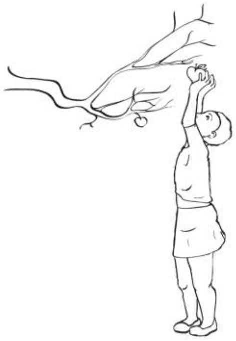

apple
bird
NoH

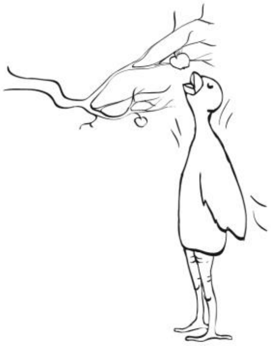

apple
bird
no-NoH

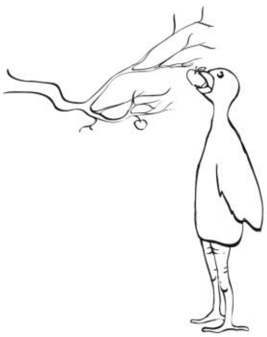

“blocks”

blocks
NoH

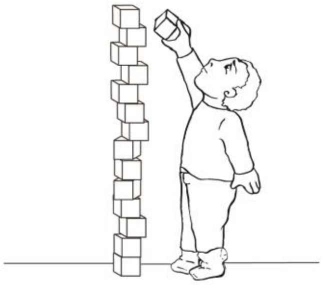

blocks
girl
NoH

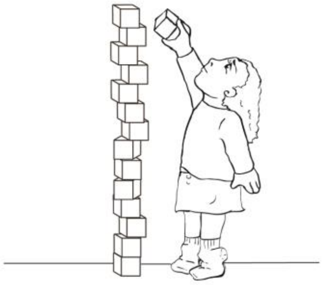

blocks
no-NoH

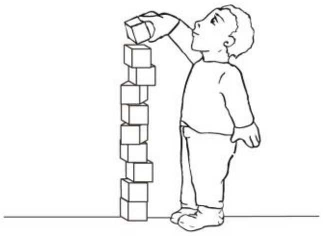

blocks
girl
no-NoH

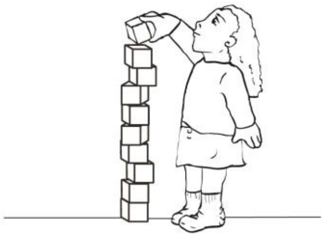

blocks
bird
NoH

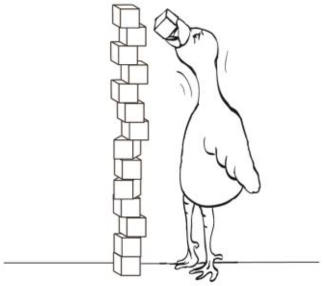

blocks
bird
no-NoH

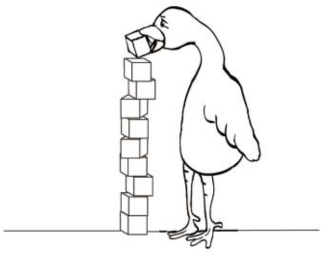

“boat”

boat
NoH

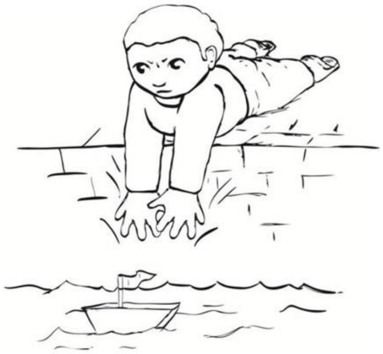

boat
girl
NoH

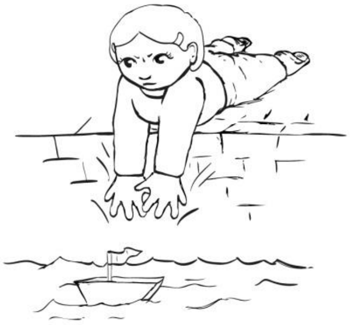

boat
no-NoH

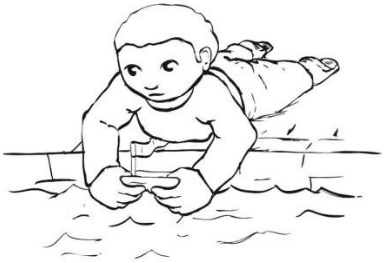

boat
girl
no-NoH

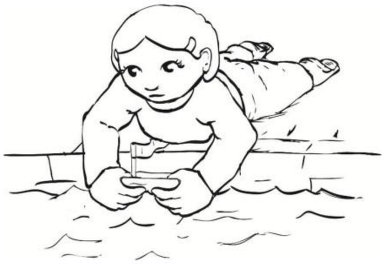

boat
bird
NoH

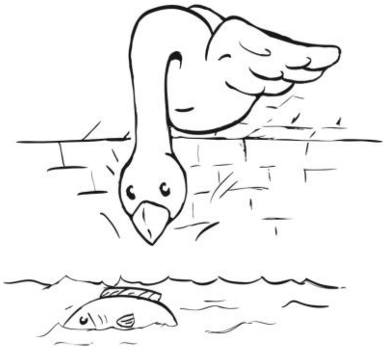

boat
bird
no-NoH

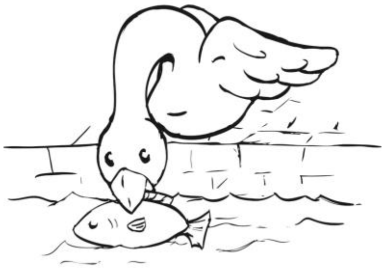

“branch”

branch
NoH

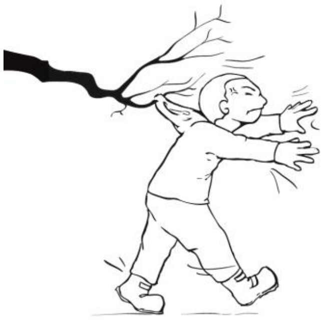

branch
girl
NoH

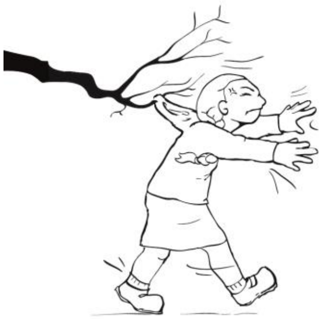

branch
no-NoH

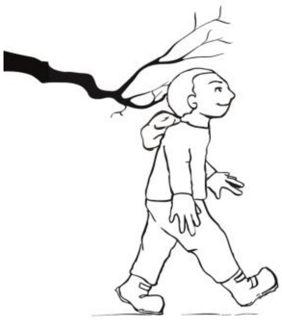

branch
girl
no-NoH

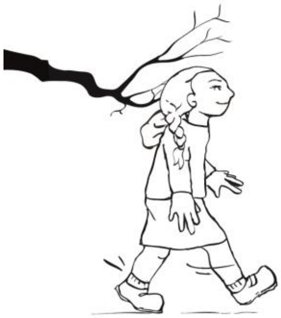

branch
bird
NoH

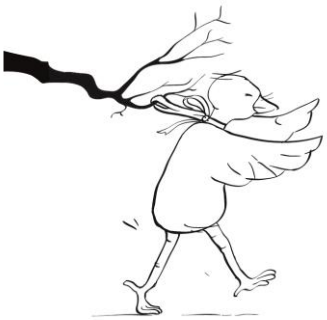

branch
bird
no-NoH

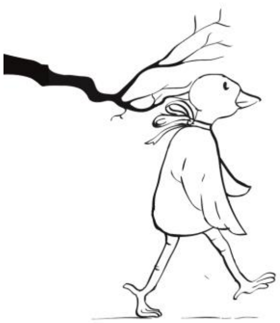

“bucket”

bucket
NoH

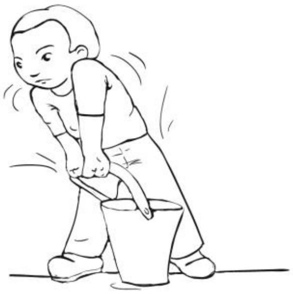

bucket
girl
NoH

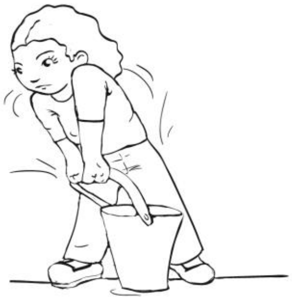

bucket
no-NoH

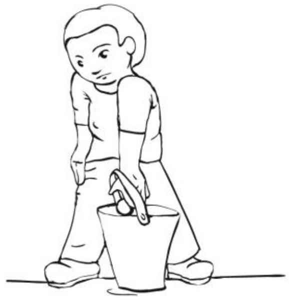

bucket
girl
no-NoH

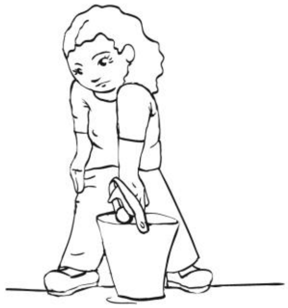

bucket
bird
NoH

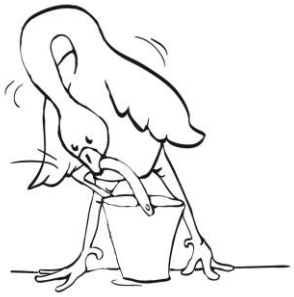

bucket
bird
no-NoH

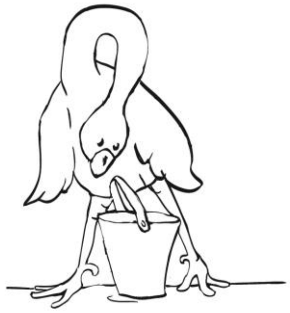

“climb”

climb
NoH

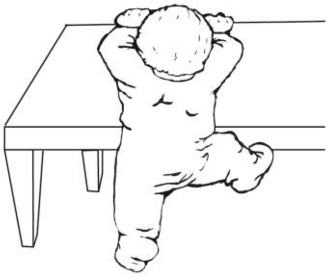

climb
no-NoH

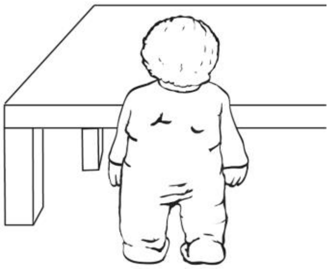

climb
bird
NoH

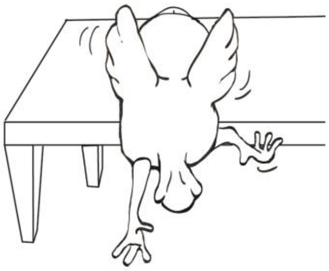

climb
bird
no-NoH

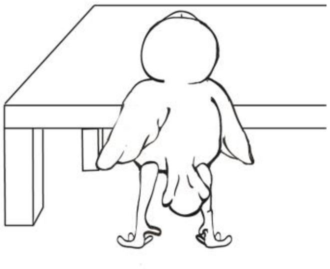

“door”

door
NoH

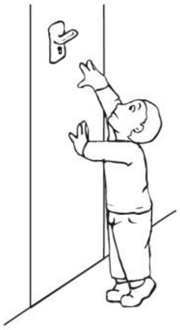

door
bird
NoH

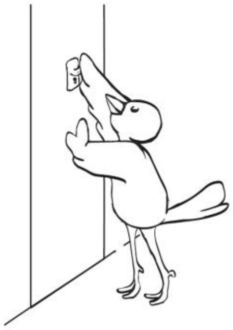

door
no-NoH

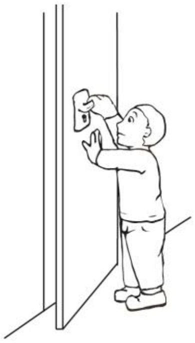

door
bird
no-NoH

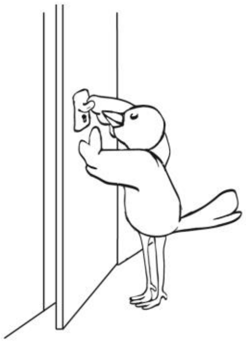

“drawer”

drawer
NoH

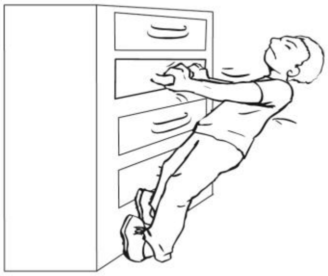

drawer
girl

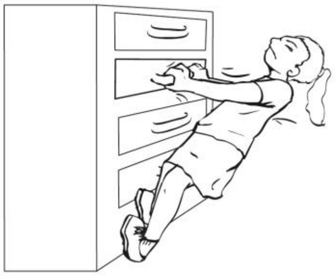

drawer
no-NoH

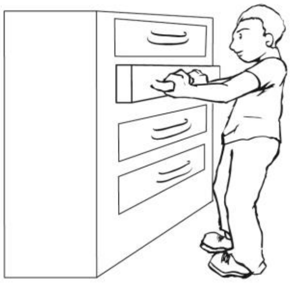

drawer
girl
no-NoH

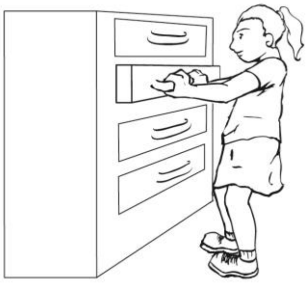

drawer
bird
NoH

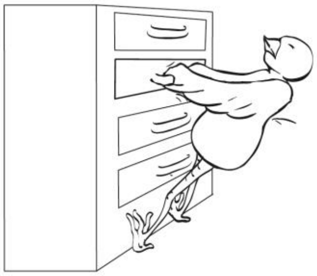

drawer
bird
no-NoH

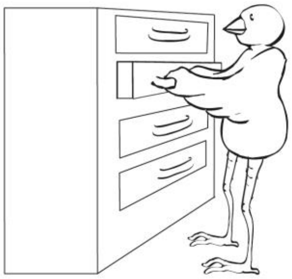

“gap”

gap
NoH

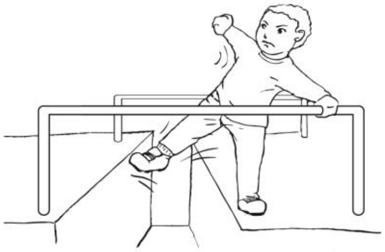

gap
girl

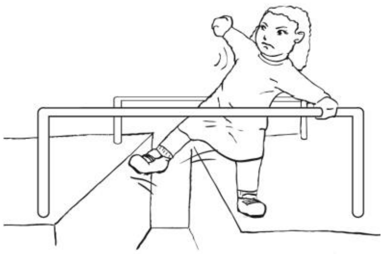

gap
no-NoH

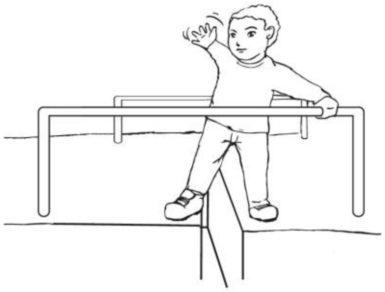

gap
girl
no-NoH

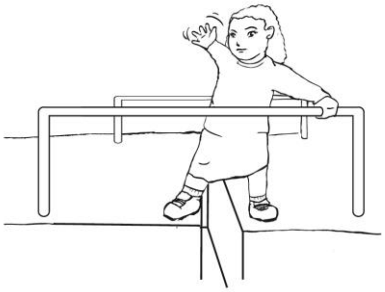

gap
bird
NoH

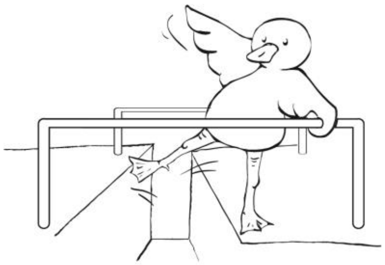

gap
bird
no-NoH

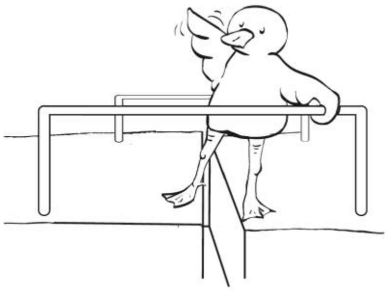

“shelf”

shelf
NoH

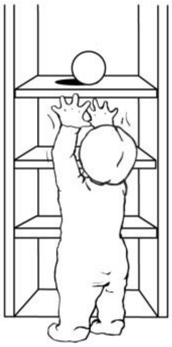

shelf
african
NoH

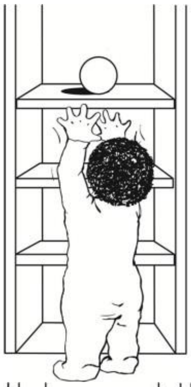

shelf
no-NoH

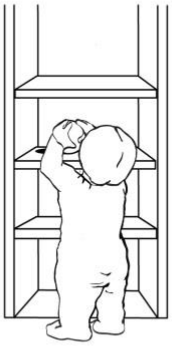

shelf
african
no-NoH

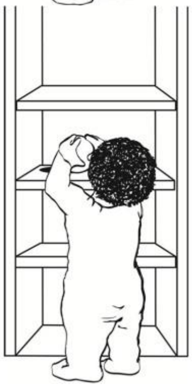

shelf
bird
NoH

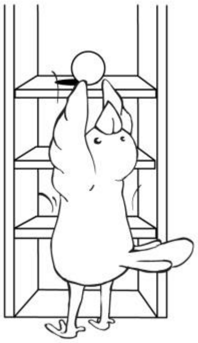

shelf
bird
no-NoH

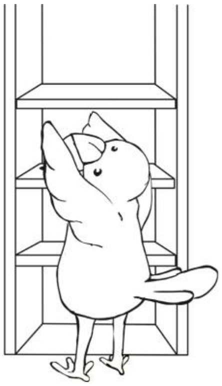

“shirt”

shirt
NoH

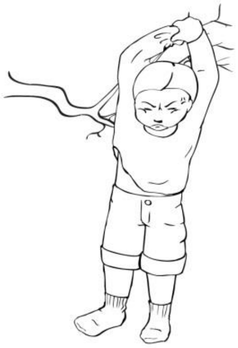

shirt
no-NoH

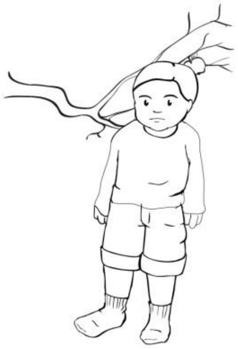

shirt
bird
NoH

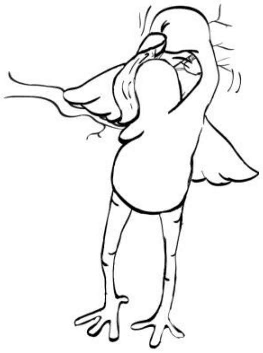

shirt
bird
no-NoH

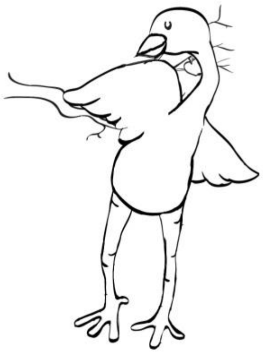

"sit"

sit
NoH

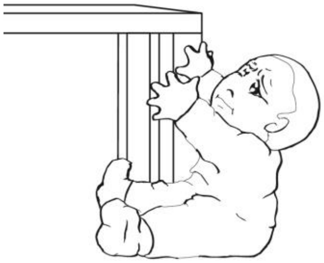

sit
bird
NoH

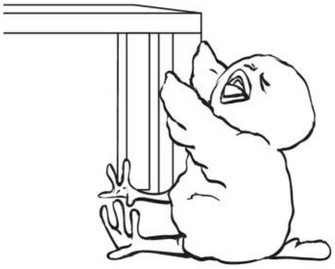

sit
no-NoH

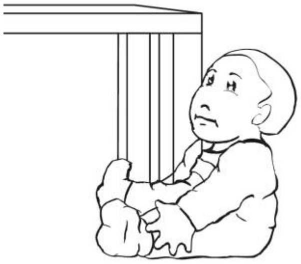

sit
bird
no-NoH

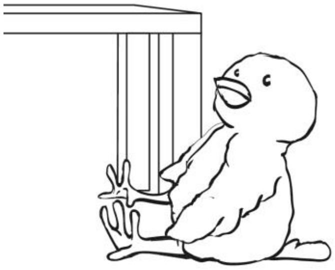

“stair”

stair
NoH

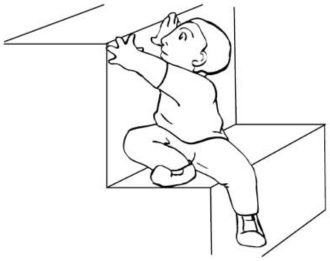

stair
girl
NoH

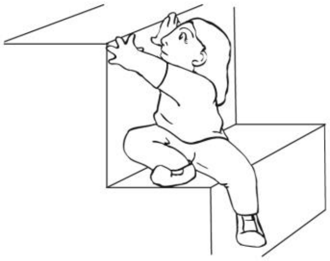

stair
no-NoH

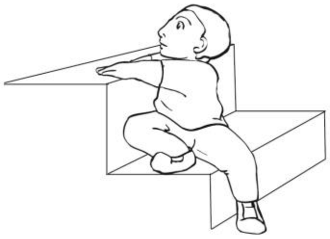

stair
girl
no-NoH

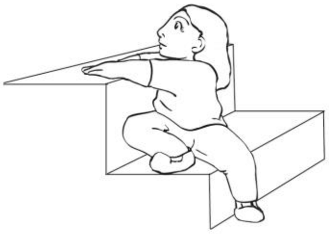

stair
bird
NoH

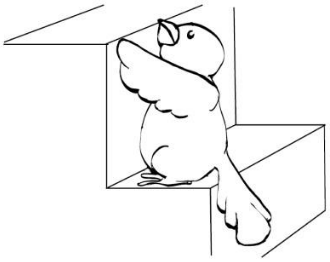

stair
bird
no-NoH

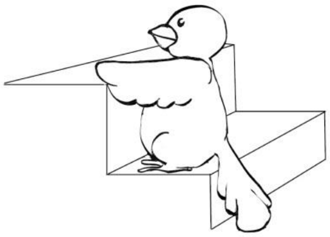

"table"

table
NoH

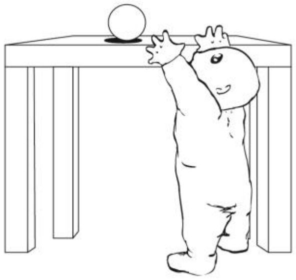

table
no-NoH

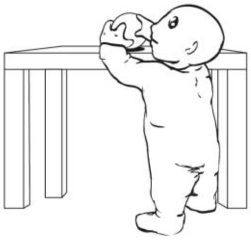

table
bird
NoH

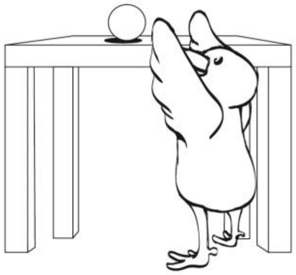

table
bird
no-NoH

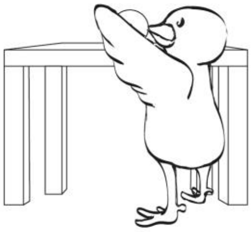

“table_chair”

table_chair
NoH

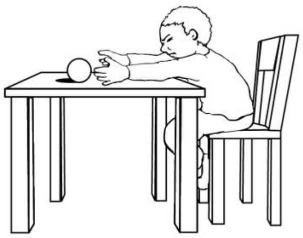

table_chair
6m
bird
NoH

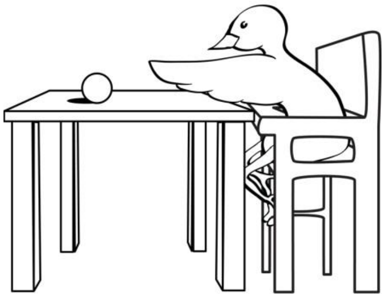

table_chair
no-NoH

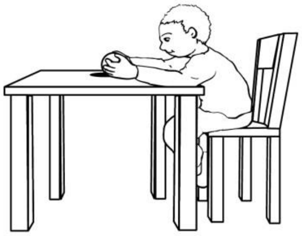

table_chair
bird
no-NoH

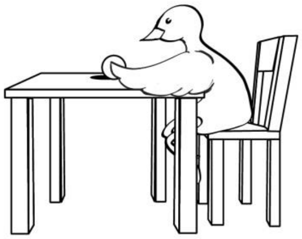

table_chair
6m
NoH

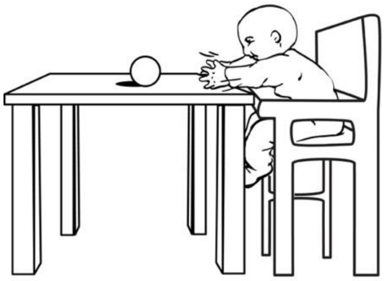

table_chair
6m
bird
NoH

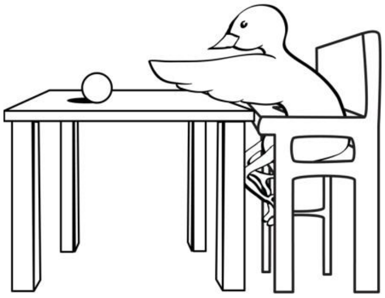

table_chair
6mbird
no-NoH

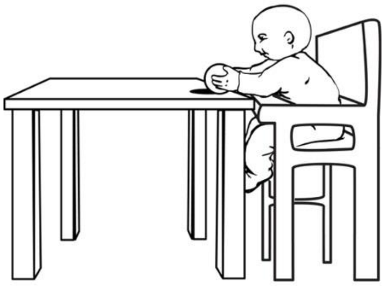

tablec_hair
6m
bird
no-NoH

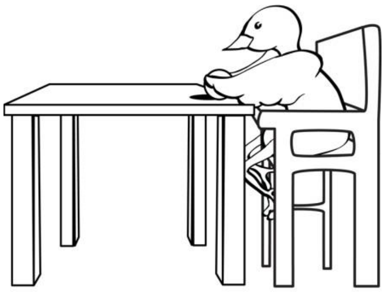

Supplement: Supplementary file 1 [file DataSheet1.PDF]
